# Incubation period of 2019 novel coronavirus (2019-nCoV) infections among travellers from Wuhan, China, 20–28 January 2020

**DOI:** 10.2807/1560-7917.ES.2020.25.5.2000062

**Published:** 2020-02-06

**Authors:** Jantien A Backer, Don Klinkenberg, Jacco Wallinga

**Affiliations:** 1 Centre for Infectious Disease Control, National Institute for Public Health and the Environment (RIVM), Bilthoven, Netherlands; 2 Department of Biomedical Data Sciences, Leiden University Medical Center, Leiden, Netherlands

**Keywords:** 2019-nCoV, novel coronavirus, incubation period, exposure, symptom onset, Wuhan

## Abstract

A novel coronavirus (2019-nCoV) is causing an outbreak of viral pneumonia that started in Wuhan, China. Using the travel history and symptom onset of 88 confirmed cases that were detected outside Wuhan in the early outbreak phase, we estimate the mean incubation period to be 6.4 days (95% credible interval: 5.6–7.7), ranging from 2.1 to 11.1 days (2.5th to 97.5th percentile). These values should help inform 2019-nCoV case definitions and appropriate quarantine durations.

Early January 2020, a novel coronavirus (2019-nCoV) was identified as the infectious agent causing an outbreak of viral pneumonia in Wuhan, China, where the first cases had their symptom onset in December 2019 [1]. This newly discovered virus, which causes severe acute respiratory disease, is related to the severe acute respiratory syndrome (SARS) coronavirus and Middle East respiratory syndrome (MERS) coronavirus, but distinct from each of these [2]. The key epidemiological parameters, including incubation period, for this new virus are therefore rapidly being studied from incoming case reports as the epidemic continues. Chief among these key parameters is the incubation period distribution. The range of the values for the incubation period is essential to epidemiological case definitions, and is required to determine the appropriate duration of quarantine. Moreover, knowledge of the incubation period helps to assess the effectiveness of entry screening and contact tracing. The distribution of the incubation period is also used in estimating the size of the epidemic [3-5] and the transmission potential [6,7]. In absence of data on the 2019-nCoV incubation period, these studies have assumed incubation periods of SARS or MERS coronaviruses.

Here we present the distribution of incubation periods estimated for travellers from Wuhan with confirmed 2019-nCoV infection in the early outbreak phase, using their reported travel histories and symptom onset dates.

## Travellers from Wuhan with confirmed 2019 novel coronavirus infection, reported symptom onset data and reported travel history

In January 2020, an increasing number of cases confirmed to be infected with 2019-nCoV were detected outside Wuhan. For 88 cases detected between 20 and 28 January, the travel history (to and) from Wuhan is known, as well as their symptom onset date. Their ages range from 2 to 72 years of age (information missing for four cases); 31 were female and 57 were male. During this initial stage of the epidemic, it is most likely that these travellers were infected in Wuhan. Consequently, their time spent in Wuhan can be taken as the duration of exposure to infection. Of these 88 cases with known travel history, 63 were Wuhan residents who travelled elsewhere and 25 were visitors who stayed in Wuhan for a limited time. By taking the date of symptom onset and travel history together, we inferred the possible incubation period for each of these cases.

The data used for this analysis has been translated from Chinese sources such as provincial centres of disease control, and made publicly available [8]. We took the data as available on 29 January 2020 (Supplementary Material S1).

## Incubation period distribution

Using the duration of stay in Wuhan and the symptom onset date, we obtained a range of possible values for the incubation period of each case. We fitted three parametric forms for the incubation period distribution to these ranges: the Weibull distribution, the gamma distribution and the lognormal distribution. We used a Bayesian approach to fitting that allows for the use of prior knowledge to inform the analysis. We specified strictly positive flat prior probability distributions for the parameter values of the three distributions (Supplementary Material S2), which ensured our estimates are conservative. Because of the sufficient number of observations, the impact of the priors on the outcome was negligible. We used a uniform prior probability distribution over the exposure interval for the moment of infection for each case. We sampled from the posterior distribution using the rstan package [9] in R software version 3.6.0 (R Foundation, Vienna, Austria) (Supplementary Material S3).

**Figure 1:**
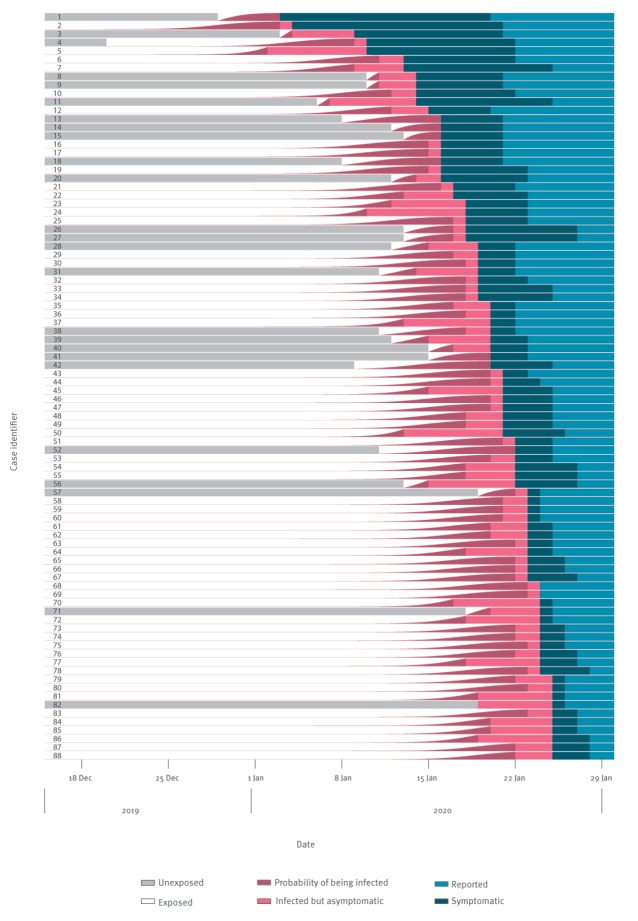
Exposure to reporting timeline for confirmed 2019 novel coronavirus (2019-nCoV) cases with travel history from Wuhan, sorted by symptom onset date, data 20–28 January 2020 (n = 88)

Figure 1 shows the exposure to reporting timeline for each case, where the cases without a maximum incubation period lack an unexposed (grey) period. However, the estimated infection times for these cases are close to the end of the exposure window, informed by the cases that do have a maximum incubation period.

The analysis yields the probability of being infected (dark pink), i.e. the cumulative density function of the estimated infection moments, using the Weibull distribution.

**Figure 2:**
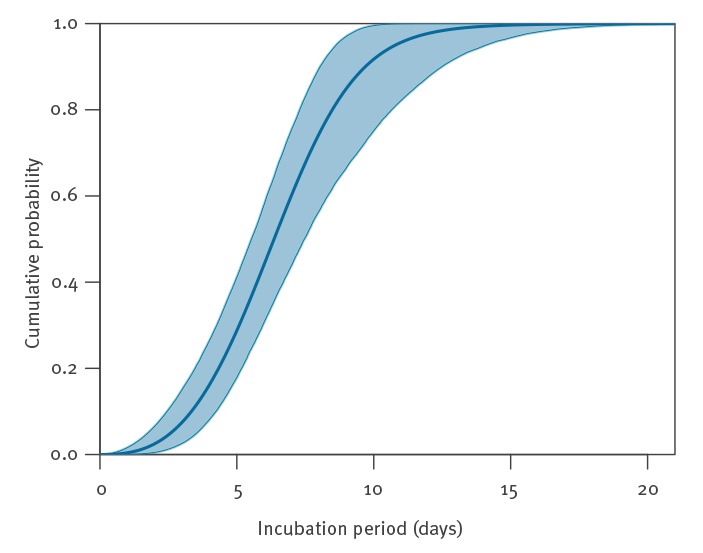
The cumulative density function of the estimated Weibull incubation period distribution for travellers infected with the 2019 novel coronavirus (2019-nCoV) in Wuhan, China, data 20–28 January 2020

The Weibull distribution provided the best fit to the data (Table 1). The mean incubation period was estimated to be 6.4 days (95% credible interval (CI): 5.6–7.7). The incubation period ranges from 2.1 to 11.1 days (2.5th to 97.5th percentile) (Table 2 and Figure 2). The results using the gamma distribution provide a slightly poorer description of the data, but a similar range: from 2.4 to 12.5 days. Although the lognormal distribution provides the poorest fit to the data, the incubation period ranging from 2.4 to 15.5 days (2.5th to 97.5th percentile) may be relevant for a conservative choice of quarantine periods.

**Table 1:** Estimated incubation period for travellers infected with 2019 novel coronavirus (2019-nCoV) in Wuhan, China, for different parametric forms of the incubation period distribution, data 20–28 January 2020

Distribution	Mean (days)		SD (days)		LOO IC^b^
	Estimate^a^	95% CI	Estimate^a^	95% CI	
Weibull	6.4	5.6–7.7	2.3	1.7–3.7	486
Gamma	6.5	5.6–7.9	2.6	1.8–4.2	545
Lognormal	6.8	5.7–8.8	3.4	2.1–6.4	592

CI: credible interval; LOO IC: Leave-one-out information criterion; SD: standard deviation.

^a ^Posterior median.

^b^ LOO IC indicates the goodness-of-fit, where lower values indicate a better fit and differences larger than two are statistically relevant.

**Table 2:** Percentiles of estimated incubation period for travellers infected with 2019 novel coronavirus (2019-nCoV) in Wuhan, China, for different parametric forms of the incubation period distribution, data 20–28 January 2020

Percentiles	Incubation period distribution (days)					
	Weibull		Gamma		Lognormal	
	Estimate^a^	95% CI	Estimate^a^	95% CI	Estimate^a^	95% CI
2.5th	2.1	1.3–3.0	2.4	1.5–3.2	2.4	1.6–3.1
5th	2.7	1.8–3.5	2.9	2.0–3.6	2.8	2.0–3.5
50th	6.4	5.5–7.5	6.1	5.3–7.3	6.1	5.2–7.4
95th	10.3	8.6–14.1	11.3	9.1–15.7	13.3	9.9–20.5
97.5th	11.1	9.1–15.5	12.5	9.9–17.9	15.5	11.0–25.2
99th	11.9	9.7–17.2	14.1	10.9–20.6	18.5	12.6–32.2

CI: credible interval.

^a ^Posterior median.

Posterior median of mean is indicated by the dark blue line and the 95% credible interval by the light blue area.

## Comparison of 2019 novel coronavirus, severe acute respiratory syndrome and Middle East respiratory syndrome coronaviruses’ incubation periods

**Figure 3:**
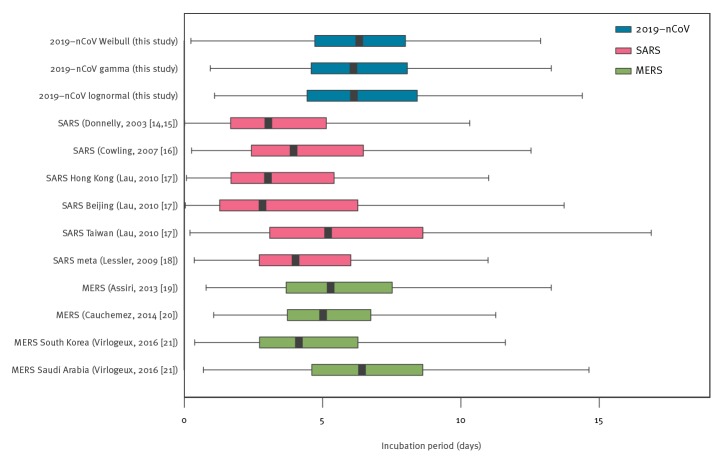
Box-and-whisker-plots of estimated incubation periods for coronaviruses from different studies

A comparison to the estimated incubation period distribution for MERS (Table 3 and Figure 3) shows that the incubation period values are remarkably similar, with mean values differing at most 1 day and 95th percentiles differing at most 2 days. The estimated mean incubation periods for SARS are more variable between studies, including values shorter and longer than those presented here for 2019-nCoV. These findings imply that the findings of previous studies that have assumed incubation period distributions similar to MERS or SARS will not have to be adapted because of a shorter or longer incubation period.

**Table 3:** Estimated incubation periods for coronaviruses from different studies

Study	Virus (location)	Distribution	Mean (days)		95^th^ percentile (days)	
			Estimate	95% CI	Estimate	95% CI
This study	2019-nCoV	Weibull	6.4	5.6–7.7	10.3	8.6–14.1
This study	2019-nCoV	Gamma	6.5	5.6–7.9	11.3	9.1–15.7
This study	2019-nCoV	Lognormal	6.8	5.7–8.8	13.3	9.9–20.5
Donnelly, 2003 [14,15]	SARS	Gamma	3.8	3.0–4.9	9.45	NA
Cowling, 2007 [16]	SARS	Lognormal	5.1	4.6–5.5	12.9	11.7–14.5
Lau, 2010 [17]	SARS (Hong Kong)	Lognormal	4.4	NA	12.4	NA
Lau, 2010 [17]	SARS (Beijing)	Lognormal	5.7	NA	19.7	NA
Lau, 2010 [17]	SARS (Taiwan)	Lognormal	6.9	NA	17.9	NA
Lessler, 2009 [18]	SARS	Lognormal	4.8^a^	3.6–4.4	10.6	8.9–12.2
Assiri, 2013 [19]	MERS	Lognormal	6.0^b^	1.9–14.7	12.4	7.3–17.5
Cauchemez, 2014 [20]	MERS	Lognormal	5.5	3.6–10.2	10.2^c^	NA
Virlogeux, 2016 [21]	MERS (South Korea)	Gamma	6.9	6.3–7.5	12.7	11.5–14.4
Virlogeux, 2016 [21]	MERS (Saudi Arabia)	Lognormal	5.0	4.0–6.6	11.4	8.5–17.5

CI: credible interval; NA: not available; 2019-nCoV: 2019 novel coronavirus; SARS; severe acute respiratory syndrome; SD: standard deviation; MERS: Middle East respiratory syndrome.

^a^ Value calculated from median of 4.0 days provided in reference.

^b^ Value calculated from median of 5.2 days provided in reference.

^c^ Value calculated from mean and SD provided in reference.

2019-nCoV: 2019 novel coronavirus; SARS; severe acute respiratory syndrome; MERS: Middle East respiratory syndrome.

Median (black point), interquartile range (box), and maximum of 1.5 times the interquartile range (whiskers).

## Discussion

We characterised the distribution of incubation periods for travellers from Wuhan infected with 2019-nCoV in Wuhan who were reported as cases between 20 and 28 January 2020. The study provides empirical evidence to back reports on a familial cluster where five family members developed symptoms 3 to 6 days after exposure [10], and fits within the range for the incubation period of 0 to 14 days assumed by the WHO and of 2 to 12 days assumed by the ECDC [11]. Our estimate of the mean incubation period is longer than the value of 5.2 days based on 10 cases [12], and 4.8 days (range: 2–11) based on 16 travellers between Wuhan and Guangdong [13]. The latter study is restricted to travellers with a 3-day exposure window. Repeating our analysis with only the 25 visitors to Wuhan who had a closed exposure window, leads to a mean incubation period of 4.5 days (CI: 3.7–5.6) which is more in line with the studies above, but the 95th percentile drops to 8.0 days (CI: 6.3–11.8).

In our analysis, we assumed a uniform prior probability of being infected during the period of stay in Wuhan. Since the epidemic was developing during that time period, it is more likely that travellers were infected towards the end rather than the beginning of their stay. This might produce a slight bias towards longer incubation periods, so the estimated upper limit of 11.1 days could be considered conservative.

The travellers in this study represent a selective sample of the reported cases. We found travellers to be more often male and younger than the cases reported in [12]. The numbers are too small to detect systematic differences in incubation time with age or sex. Because we only have information on confirmed cases, there is likely a bias towards more severe cases in areas with early awareness and a well-functioning healthcare system. As the epidemic continues, it remains important to collect more information on the incubation periods of 2019-nCoV cases with older ages, with underlying morbidity, who are women or who have mild symptoms.

There are various choices one can make about the parametric form of the incubation period distribution. The results with the three often-used forms we report here suggest that there is little impact on the mean and dispersion of the incubation periods. Of these three, the lognormal distribution assigns higher probabilities to longer incubation periods. Although we found that this distribution provided a poorer description of the data than the Weibull and the gamma distributions, it is prudent not to dismiss the possibility of incubation periods up to 14 days at this stage of the epidemic.

## Supplementary Data

528000 Supplementary Material
