# Supplementary material for: Incubation period of 2019 novel coronavirus (2019-nCoV) infections among travellers from Wuhan, China, 20–28 January 2020
Source: Euro Surveill. 2020 Feb 6;25(5):2000062. doi: 10.2807/1560-7917.ES.2020.25.5.2000062 (PMC7014672; doi:10.2807/1560-7917.ES.2020.25.5.2000062)
Supplement: Supplementary Material [file 20-00062_BAKER_SupplementMaterial.zip › 20-00062_BAKER_SupplementMaterial/BAKER_Supplementary material DISCLAIMER.pdf]

Supplementary Material

"This supplementary material is hosted by *Eurosurveillance* as supporting information alongside the article [Incubation period of 2019 novel coronavirus (2019-nCoV) infections among travellers from Wuhan, China, 20–28 January 2020], on behalf of the authors, who remain responsible for the accuracy and appropriateness of the content. The same standards for ethics, copyright, attributions and permissions as for the article apply. Supplements are not edited by *Eurosurveillance* and the journal is not responsible for the maintenance of any links or email addresses provided therein."
