# Supplementary material for: Incubation period of 2019 novel coronavirus (2019-nCoV) infections among travellers from Wuhan, China, 20–28 January 2020
Source: Euro Surveill. 2020 Feb 6;25(5):2000062. doi: 10.2807/1560-7917.ES.2020.25.5.2000062 (PMC7014672; doi:10.2807/1560-7917.ES.2020.25.5.2000062)
Supplement: Supplementary Material [file 20-00062_BAKER_SupplementMaterial.zip › 20-00062_BAKER_SupplementMaterial/BAKER_Supplementary material S2_parametrisation.pdf]

This supplementary material is hosted by Eurosurveillance as supporting information alongside the article "The incubation period of 2019-nCoV infections among travellers from Wuhan, China", on behalf of the authors, who remain responsible for the accuracy and appropriateness of the content. The same standards for ethics, copyright, attributions and permissions as for the article apply. Supplements are not edited by Eurosurveillance and the journal is not responsible for the maintenance of any links or email addresses provided therein.

S2
Parametrisation of incubation period distriubtions and prior information

Weibull distribution

Weibull(λ, k) with mean $\lambda\Gamma(1+1/k)$ and variance $\lambda^2\left[\Gamma(1+2/k) - \Gamma(1+1/k)^2\right]$
using strictly positive, flat prior probabilites on scale parameter λ and shape parameter k.

Gamma distribution

Gamma(k, θ) with mean $k\theta$ and variance $k\theta^2$
using strictly positive, flat prior probabilites on the mean $k\theta$ and shape parameter k.

Lognormal distribution

Lognormal(μ, σ) with mean $\exp(\mu + \sigma^2/2)$ and variance $\left[\exp(\sigma^2 - 1\right] \exp(2\mu + \sigma^2)$
using strictly positive, flat prior probabilites on the logmean μ and logsd σ.
